# Socioeconomic and geographic disparities in psychiatric outcomes under Colombia’s universal healthcare system

**DOI:** 10.1017/S0033291725101694

**Published:** 2025-10-13

**Authors:** Greta Gerdes, Janet Song, Susan K. Service, Ana M. Ramirez-Diaz, Ana M. Diaz-Zuluaga, Alejandro Arias, Mauricio Castaño-Ramirez, Nicolas A. Crossley, Carlos Lopez-Jaramillo, Nelson B. Freimer, Loes M. Olde Loohuis

**Affiliations:** 1 Center for Neurobehavioral Genetics, Semel Institute for Neuroscience and Human Behavior, University of California Los Angeles, Los Angeles, CA, USA; 2 Northwestern University, Feinberg School of Medicine, Chicago, IL, USA; 3 Department of Psychiatry, University of Antioquía, Medellín, Colombia; 4 Department of Mental Health and Human Behavior, Universidad de Caldas, Manizales, CAL, Colombia; 5 Department of Psychiatry, University of Oxford, Oxford, UK; 6 Department of Psychiatry, Pontificia Universidad Católica de Chile, Santiago, Chile; 7 Department of Human Genetics, David Geffen School of Medicine, University of California Los Angeles, Los Angeles, CA, USA; 8 Department of Computational Medicine, David Geffen School of Medicine, University of California Los Angeles, Los Angeles, CA, USA

**Keywords:** access to healthcare, electronic health records, mood and psychotic disorders, socioeconomic status, social support, travel time

## Abstract

**Background:**

Despite growing healthcare coverage, disparities in access to and outcomes of psychiatric care persist, even in countries with universal healthcare. How socioeconomic status (SES), travel time, and social support individually and jointly affect psychiatric clinical trajectories remains largely unexplored.

**Methods:**

We analyze electronic health records (EHRs) from patients diagnosed with bipolar disorder, major depressive disorder, or schizophrenia at Clínica San Juan de Dios Manizales. Using zero-inflated and standard negative binomial regression, we quantify the effects of SES, travel time, and family/social support on utilization, clinical outcomes, and symptoms of mania, psychosis, and suicidality. A mixed-effects model examines how care-seeking patterns affect visit-to-visit variability in outcomes.

**Results:**

Among 21,095 patients, utilization is lower for those with low SES (rate ratio [RR] 0.92, 95% CI: 0.90–0.95, *p* = 1.27e−10) and longer travel times (RR 0.94, 95% CI: 0.93–0.95, *p* = 1.19e−53). Patients with low SES are more likely to have severe symptoms (e.g., delusions: RR 1.28, 95% CI: 1.20–1.37, *p* = 2.57e−15) and require hospitalization (RR 1.10, 95% CI: 1.05–1.15, *p* = 1.94e−04), suggesting they primarily seek care when critical. Longer travel differentially affects those with low SES. However, the relationship between SES and adverse outcomes is less pronounced when living with family (e.g., hospitalizations: LRT, *χ*^2^ = 47.08, df = 3, *p* = 3.35e−10). Frequent outpatient care is associated with lower odds of hospitalization, suicidality, and other symptoms.

**Conclusions:**

Findings demonstrate use of EHRs to model patient outcomes, the important role of social support, and need for improved healthcare accessibility.

## Introduction

Over the past two decades, studies estimate that more than half of individuals worldwide with psychiatric disorders go untreated, even in countries with universal healthcare (Kohn et al., 2018; Kohn, Saxena, Levav, & Saraceno, 2004; Patel et al., 2010; The WHO World Mental Health Survey Consortium, 2004). This treatment gap is due, in part to, geographic barriers like proximity to healthcare facilities (Cummings, Allen, Clennon, Ji, & Druss, 2017; Evans et al., 2022; Labban et al., 2023; Negaro et al., 2023; Syed, Gerber, & Sharp, 2013; Wallace, Hughes-Cromwick, Mull, & Khasnabis, 2005) and social and systemic factors, including socioeconomic status (SES), insurance coverage, cultural or language barriers, social stigma, and disparities based on ethnicity, race, and gender (Báscolo, Houghton, & Del Riego, 2020; Caraballo et al., 2022; Daher et al., 2021; Evans-Lacko et al., 2018; Kim, Vonneilich, Lüdecke, & von dem Knesebeck, 2017; Kohn et al., 2018; Lazar & Davenport, 2018; Rahman et al., 2022; Sentell, Shumway, & Snowden, 2007; Thornicroft et al., 2009).

To address inequities in access to care, many Latin American countries implemented healthcare reforms starting in the 1980s (Atun et al., 2015; Göttems & Mollo, 2020). In 1993, Colombia introduced universal healthcare under the General System of Social Security in Health (SGSSS) (Alvarez, Salmon, & Swartzman, 2011), significantly improving access to preventative care (Garcia-Ramirez, Nikoloski, & Mossialos, 2020; Miller, Pinto, & Vera-Hernández, 2013). This system includes two main insurance schemes: the subsidized scheme, which is state funded for individuals with limited financial means, and the contributory scheme, which is for those who can afford to contribute a portion of their income toward insurance. Colombia uses the Sistema de Identificación de Potenciales Beneficiarios de Programas Sociales (SISBÉN) to determine eligibility for subsidized insurance based on income, housing, education, and demographic information (Departamento Nacional de Planeación (DNP), n.d.-a; International Labour Office, 2015). While exact income criteria are not specified, individuals who are unemployed, living in poverty, or unable to afford contributory insurance are eligible for subsidized coverage. Accordingly, insurance status can serve as a proxy for SES in Colombia (Carabali, Schmidt, Restrepo, & Kaufman, 2022; Guarnizo-Herreño, Torres, & Buitrago, 2021; Viáfara-López, Palacios-Quejada, & Banguera-Obregón, 2021).

Despite near-universal insurance coverage in Colombia (Brun Vergara, Garcia Ruiz, & Guzman, 2023), care-seeking patterns and psychiatric outcomes may still vary due to barriers such as travel time, SES, or a combination of factors. In previous work, Song et al. used electronic health records (EHRs) from Clínica San Juan de Dios Manizales (CSJDM) to investigate geographic variation in incidence of severe mood and psychotic disorders in Caldas, Colombia (Song et al., 2024). CSJDM, located in the metropolitan municipality of Manizales, serves as the primary psychiatric care provider for the department (state) of Caldas, which has nearly one million residents. Song et al. found fewer-than-expected outpatient cases in areas farther from the hospital, while inpatient cases remained stable, suggesting that travel time restricts access to non-emergent care.

Existing research on the link between SES and psychiatric illness, while extensive, has primarily examined the incidence of psychiatric illness across demographic groups using cross-sectional data (Carlborg, Ferntoft, Thuresson, & Bodegard, 2015; Freeman et al., 2016; Hakulinen, Musliner, & Agerbo, 2019; Kivimäki et al., 2020; Qin et al., 2008; Sletved, Ziersen, Andersen, Vinberg, & Kessing, 2023). In contrast, research on how clinical outcomes are impacted by SES and related barriers is sparse. Most studies in this domain focused on a single psychiatric outcome and/or relied on surveys or self-report questionnaires (Al-Otaibi et al., 2007; Angstman, Wi, Williams, Bohn, & Garrison, 2021; Buckman et al., 2022; Cwikel, Zilber, Feinson, & Lerner, 2008; Knudsen, Valentin, Videbech, Mainz, & Johnsen, 2022; Lu et al., 2020; Madigan & Daly, 2023; Schlax et al., 2019; Sletved et al., 2022), which can be subject to response bias and limited sample sizes. Moreover, although strong family/social support is linked to better treatment adherence and outcomes (DiMatteo, 2004; Dou et al., 2021; Giannelis et al., 2021; Hendryx, Green, & Perrin, 2009; Molarius et al., 2009; Semahegn et al., 2020; Wang, Mann, Lloyd-Evans, Ma, & Johnson, 2018), it is unclear how this support helps patients to navigate geographic and socioeconomic barriers to care.

Consequently, the individual and joint effects of SES, travel time, and social support on care-seeking patterns and subsequent psychiatric outcomes remain largely unexplored. We addressed this gap using EHRs from CSJDM (Song et al., 2024), a comprehensive longitudinal dataset containing healthcare utilization, clinical outcome, and patients’ social and demographic information for all individuals receiving care. In this study, we first examined the SES distribution of patients relative to the local population to better understand the demographics of individuals seeking care. Then, we quantified the effects of SES and travel time on healthcare utilization, clinical outcomes, including hospitalizations, ER visits, suicide attempts, and symptoms of mania, psychosis, and suicidal ideation. We evaluated interaction effects to determine whether travel time differentially impacts patients with low and higher SES. We also examined household composition to determine whether having family/social support impacts access to care and outcomes. Finally, using a longitudinal approach, we analyzed how recent outpatient and inpatient care and recent changes in SES affect visit-to-visit variability in symptoms and outcomes.

## Methods

### Ethical approval

This study was approved by the Institutional Review Boards at the University of California, Los Angeles (UCLA), Universidad de Antioquia (UdeA), and Clínica San Juan de Dios Manizales (CSJDM).

### EHR information

We leveraged EHRs from CSJDM, a large psychiatric hospital that offers outpatient care, inpatient hospitalization, and emergency services. The capture area of CSJDM was well-defined, as it serves as the primary provider of psychiatric services in Caldas. From patient records, spanning 2005 (start of EHR) to 2020, we used clinical notes, diagnostic codes, demographic information, residential addresses, and insurance information. The diagnostic codes in the EHR are reported using the International Statistical Classification of Diseases and Related Health Problems: 10th Revision (ICD-10) (World Health Organization, 2004). These diagnostic codes are congruent with diagnoses obtained through manual chart review of CSJDM EHR by psychiatrists (De la Hoz et al., 2025).

### Cohort definition

The cohort included patients diagnosed with bipolar disorder (BD, ICD-10: F31X), major depressive disorder (MDD, F32X or F33X), or schizophrenia (SCZ, F20X) at their most recent hospital/clinic encounter. We included patients ages 18–90 (age at diagnosis) residing in Caldas with complete demographic information and at least one clinical note. Sex was designated based on self-report in the EHR.

### Outcomes and EHR-derived predictors

Healthcare utilization was defined as a patient’s total number of visits at CSJDM, modeled as a rate over years in the EHR system. For each visit, we extracted symptom information (delusions, hallucinations, grandiosity, and suicidal ideation) and insurance from clinical notes. The Natural Language Processing (NLP) pipeline used was shown to accurately and reliably extract symptom and behavior data from CSJDM clinical notes (De la Hoz et al., 2025).

In alignment with SISBÉN classification (Rodríguez, Silva, & Zapata, 2024), we used insurance as a proxy for SES and classified patients with subsidized insurance as having low SES and those with contributory insurance as having higher SES, comparatively. Although insurance is recorded at each visit, we designated patients’ SES according to their most frequently listed type, unless otherwise specified.

Household composition, recorded at first intake, was used as a proxy for family and social support. It was categorized as: (1) living with parents, siblings, and/or extended family; (2) living with a partner and/or child/ren; (3) living alone; or (4) missing or other. Compositions that did not fit into the first three categories (e.g., living with a friend) were grouped with missing data, as these cases were rare, comprising about 2% of patients.

Education level, recorded in the same way, was categorized as: (1) basic education, covering grades 1 through 9; (2) upper secondary, covering grades 10 and 11; (3) technical degrees, typically lasting 2–4 years; (4) higher education, including bachelor’s, master’s, or doctoral degrees; and (5) missing.

### Geocoding procedure

Geocoding was run locally using the R package ggmap v4.0.0 (Kahle & Wickham, 2013), which takes an address as input and returns the corresponding longitude and latitude coordinates using the Google Maps Platform API (Google Maps Platform, n.d.). Before geocoding, we standardized the address format to align with the Colombian postal system (Supplemental Methods). Only locations correctly identified within the boundaries of the municipality listed in the EHR were included to ensure accuracy.

### Calculating travel time to CSJDM

To estimate each patient’s travel time to CSJDM, we used a geographic accessibility map of Caldas, which calculated the minimum travel time across one-square-kilometer grids to reach the hospital, based on topography, land cover, water bodies, and road networks. Patient coordinates were overlaid onto the map to extract individual travel times. For details on map construction, see Song et al. (2024).

### Statistical analysis

Statistical models are described below, with model equations, methods for estimating confidence intervals and significance, multiple testing correction, and additional details in the Supplemental Methods. For models with categorical variables, the reference group was the category with the largest sample size, unless specified otherwise.

#### Comparing the socioeconomic distribution of patients to the general population

To compare the SES distribution of the patient cohort to that of the Caldas population, we conducted an exact binomial test. Estimates of low SES (subsidized insurance) within the general population were obtained from Colombia’s National Department of Planning (DNP) for each municipality, based on 2018 data (Departamento Nacional de Planeación (DNP), n.d.-b). We compared the proportion of low-SES patients to the broader population of Caldas, stratified by diagnosis group and municipality.

#### Modeling the effect of travel time and SES on healthcare utilization, symptoms, and clinical outcomes

To model health care utilization, we used negative binomial regression to account for overdispersion in visit counts. This overdispersion indicates that while most patients had few visits, some patients had numerous visits during their treatment. We adjusted for age, sex, social support (household composition), diagnosis, year of the diagnosis, and diagnostic switches between BD, MDD, or SCZ (as these may influence healthcare utilization). We also adjusted for the number of hospitalizations, as illness severity may affect visit frequency. Lastly, we included an offset for the number of years a patient has been in the EHR to model visits as a rate per year.

We then modeled the effects of travel time and SES on the four symptoms (mentions of delusions, hallucinations, grandiosity, and suicidal ideation) and three clinical outcomes (number of hospitalizations, ER visits, and suicide attempts) using zero-inflated negative binomial regression to account for overdispersion in outcome and symptom counts. This overdispersion reflects that while most patients had few or no recorded delusions, for example, some patients had many. Travel time and SES were modeled jointly, adjusting for the same covariates mentioned above. An offset was included to model instances of the outcomes/symptoms per total visits.

Since 66% of patients reside in the large metropolitan municipality of Manizales, we performed a sensitivity analysis by separating patients from Manizales and other municipalities to ensure urban residency did not drive the observed SES effects. As an additional sensitivity analysis, we included education level as a covariate to assess whether the effects of travel time and socioeconomic status remained robust after adjusting for potential confounding.

#### Modeling interaction effects between travel time and SES

To assess whether patients with low SES are differentially affected by travel time compared to patients with higher SES, we extended the negative binomial model of healthcare utilization and the zero-inflated negative binomial models of clinical outcomes/symptoms (Approach 2) to include an additional travel time–SES interaction term.

#### Modeling interaction effects of household composition with SES and travel time

Since nearly 49% of the cohort had missing household composition data, we first conducted a logistic regression to identify demographic factors associated with missingness, including travel time, SES, age, sex, diagnosis, presence of a diagnosis switch, and year of diagnosis. For models involving household composition, patients living with a partner and/or child/ren were the reference group.

We then extended the models described in Approach 2 to include interaction terms for travel time–household composition and SES–household composition to assess how the effects of household composition vary by both travel time and SES.

#### Modeling visit-to-visit variability in symptoms and outcomes

We used mixed-effects logistic regression to model how changes in SES and hospitalization and outpatient care in the last 2 months affected patients’ visit-specific states. Each clinical outcome and symptom was modeled as a binary variable at the visit level. A patient-specific ID as well as visit year were included as random intercepts to account for repeated visits and hospital-level trends across years. SES changes were categorized as transitions from low to higher SES or vice versa. Models were adjusted for travel time, SES at visit, age, sex, diagnosis, and diagnostic changes since the last visit.

## Results

**Figure 1.**
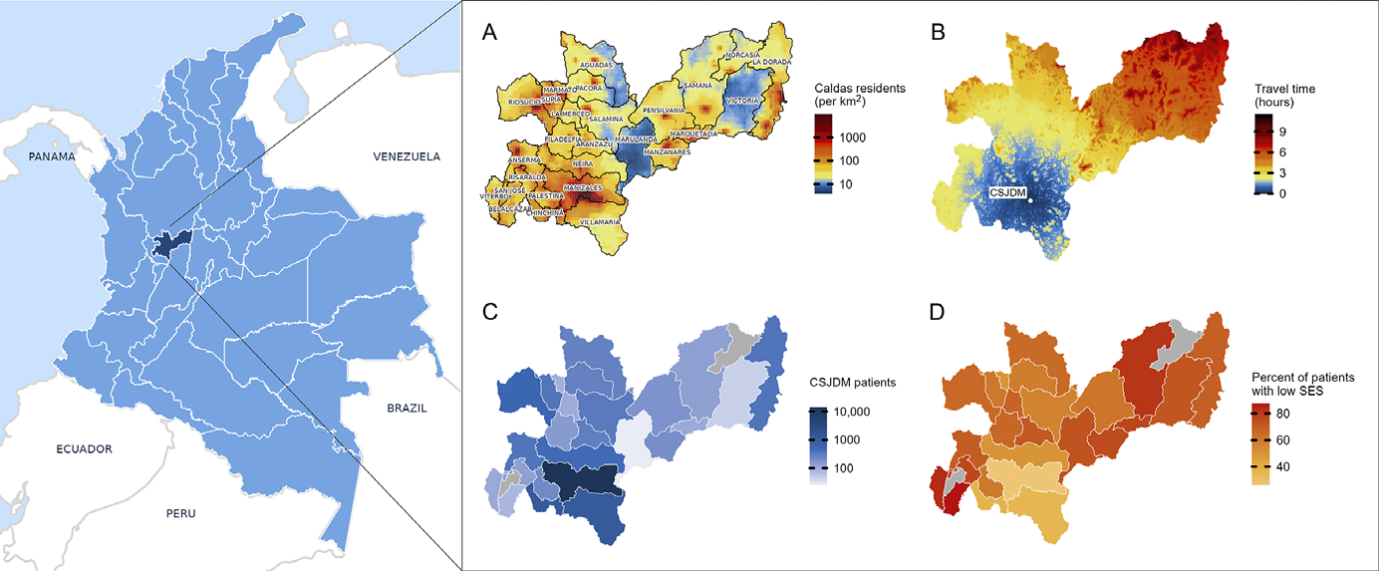
Population statistics, healthcare access, and patient characteristics in Caldas, Colombia. (a) Population density (persons per square km) with administrative boundaries labeled. (b) Travel time (hours) to Clínica San Juan de Dios Manizales (CSJDM) from any location in Caldas. (c) Number of patients residing in each municipality. (d) Percent of patients with low SES (subsidized insurance) per municipality. Grey shading indicates no patients from that municipality.

Between 2005 and 2020, the EHR system contained data for 87,870 patients, of whom 26,877 patients were adults diagnosed with BD, MDD, or SCZ and had at least one clinical note. After excluding patients without geocoded locations (*n* = 2,787) and insurance information (*n* = 2,995), the final cohort included 21,095 individuals. Table 1 summarizes cohort demographics, stratified by SES and travel time. MDD was the most common diagnosis (56%), followed by BD (36%) and SCZ (8%). Figure 1 displays characteristics of both Caldas and the study cohort. Most patients (66%) lived in Manizales, where 95% could reach the hospital within 15 minutes by car. Those outside Manizales traveled an average of 2 hours. Municipalities distant from Manizales had higher proportions of patients with low SES.

**Table 1.** Demographic and clinical characteristics by socioeconomic status and travel time *Percentages are calculated across rows within each category (socioeconomic status and travel time)

	Total	Patients, No. (%)			
		Socioeconomic Status		Travel Time	
		Low SES	Higher SES	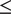 2 hours	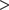 2 hours
	21095	7667 (36)	13428 (64)	17529 (83)	3566 (17)
**Diagnosis**					
BD	7698	3318 (43)	4380 (57)	6151 (80)	1547 (20)
MDD	11724	3254 (28)	8470 (72)	10136 (86)	1588 (14)
SCZ	1673	1095 (65)	578 (35)	1242 (74)	431 (26)
**Age, years**					
18–30	4987	1822 (37)	3165 (63)	4203 (84)	784 (16)
31–50	7011	2453 (35)	4558 (65)	5840 (83)	1171 (17)
51–70	7035	2648 (38)	4387 (62)	5747 (82)	1288 (18)
71–90	2062	744 (36)	1318 (64)	1739 (84)	323 (16)
**Sex**					
Female	13387	4606 (34)	8781 (66)	11260 (84)	2127 (16)
Male	7708	3061 (40)	4647 (60)	6269 (81)	1439 (19)

### Underrepresentation of low SES individuals diagnosed with MDD in the hospital system

Only 36% of the study patients have low SES, with an even lower proportion among MDD patients (28%) (Table 1). Compared to the general population of Caldas, MDD patients with low SES were significantly underrepresented in the hospital system (observed: 28%, expected: 40%, *P* = 9.56e−178), especially in rural areas (Supplemental Figure 1). In contrast, patients diagnosed with BD (observed: 43%, expected: 40%, *P* = 8.38e−07) and SCZ (observed: 65%, expected: 40%, *P* = 3.85e−95) were more likely to have low SES compared to the general population, consistent with prior research (Agerbo et al., 2015; Hakulinen et al., 2019; Kivimäki et al., 2020).

### Socioeconomic and geographic disparities in healthcare utilization and clinical outcomes

**Figure 2.**
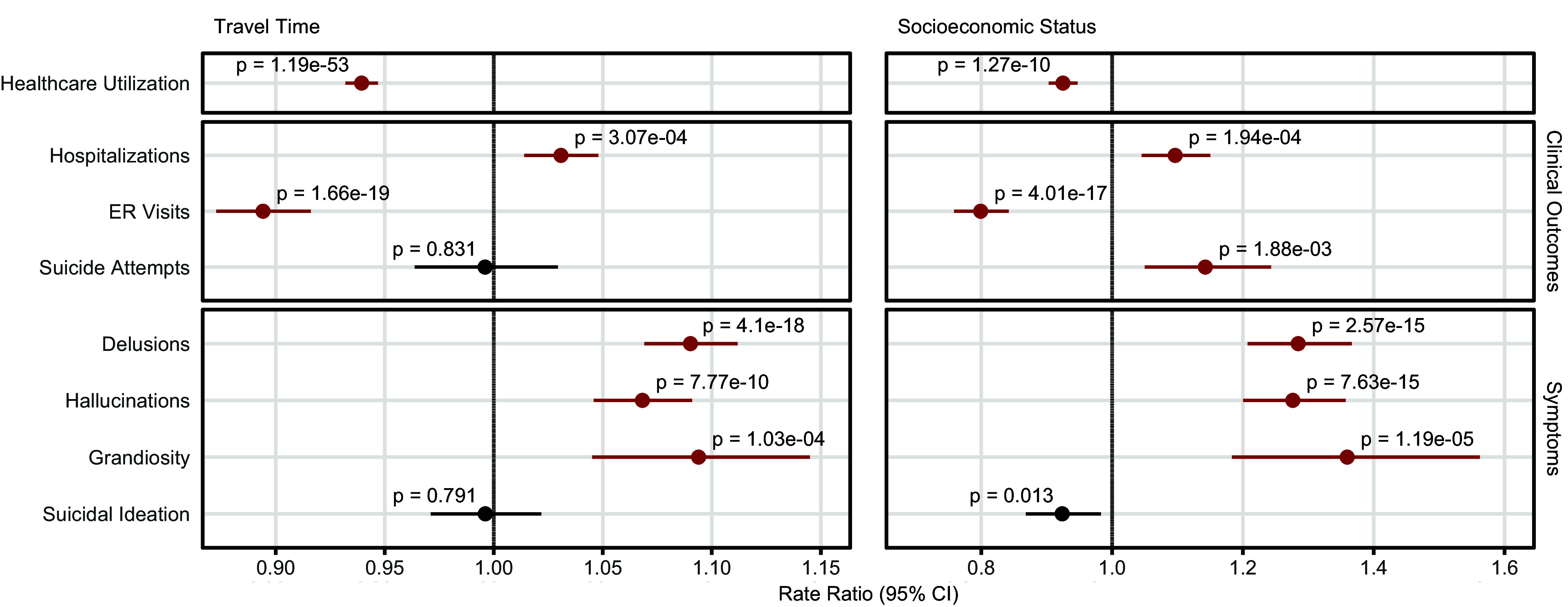
Effects of travel time and SES on healthcare utilization, clinical outcomes, and symptoms. Rate ratios are shown with bootstrap 95% confidence intervals and *p*-values. Effects from healthcare utilization models were estimated using negative binomial regression, and outcome/symptoms counts were estimated using zero-inflated negative binomial regression. Significant results, based on the Bonferroni correction threshold of 3.57e-03 (0.05/14 tests), are highlighted in red. SES reference = higher SES group.

Healthcare utilization rates varied by both SES and travel time (Figure 2). Specifically, patients with low SES were estimated to have 8% fewer visits per year, compared to those with higher SES (RR, 0.92 [95% CI, 0.90–0.95], *P* = 1.27e−10). Each additional hour of travel time was associated with a 6% reduction in visit frequency (RR, 0.94 [95% CI, 0.93–0.95], *P* = 1.19e−53), corresponding to over 30% lower rates of utilization for those living more than 5 hours away. These findings aligned with those reported by Song et al. (2024). The full model summary can be seen in Supplemental Table 1.

Patients with low SES or longer travel times tended to seek care primarily when their condition was severe, having higher rates of hospital admission and symptoms of mania/psychosis. Patients with low SES had 28% higher rates of delusion (RR, 1.28 [95% CI, 1.21–1.37], *P* = 2.57e−15), 28% higher rates of hallucinations (RR, 1.28 [95% CI, 1.20–1.36], *P* = 7.63e−15), and 36% higher rates of grandiosity (RR, 1.36 [95% CI, 1.18–1.56], *P* = 1.19e−05), even when accounting for diagnosis. They were also predicted to have 10% higher rates of hospital admission compared to patients with higher SES (RR, 1.10 [95% CI, 1.05–1.15], *P* = 1.94e−04), while their rates of ER usage were 20% lower (RR, 0.80 [95% CI, 0.76–0.84], *P* = 4.01e−17). Sensitivity analyses indicated that the SES effects on hospital admissions and ER use were largely driven by proximity to the hospital, whereas effects on symptoms were more consistent across different travel times.

Travel time had more modest effects on rates of hospital admission, ER usage, and symptoms of psychosis and mania (Figure 2). For every 1-hour increase in travel time, hospital admission rates increased by 3% (RR, 1.03 [95% CI, 1.01–1.05], *P* = 3.07e−04), while the rate of ER visits decreased by 11% (RR, 0.89 [95% CI, 0.87–0.92], *P* = 1.66e−19). For every 1-hour increase in travel time, rates of psychosis/mania-related symptoms all increased by over 5%. A full summary of zero-inflated negative binomial models can be found in Supplemental Table 2.

Sensitivity analysis revealed that the effects of travel time and socioeconomic status are unchanged after accounting for education level, available in 46% of patients (data not shown).

### Patients with low SES are differentially affected by longer travel times

**Figure 3.**
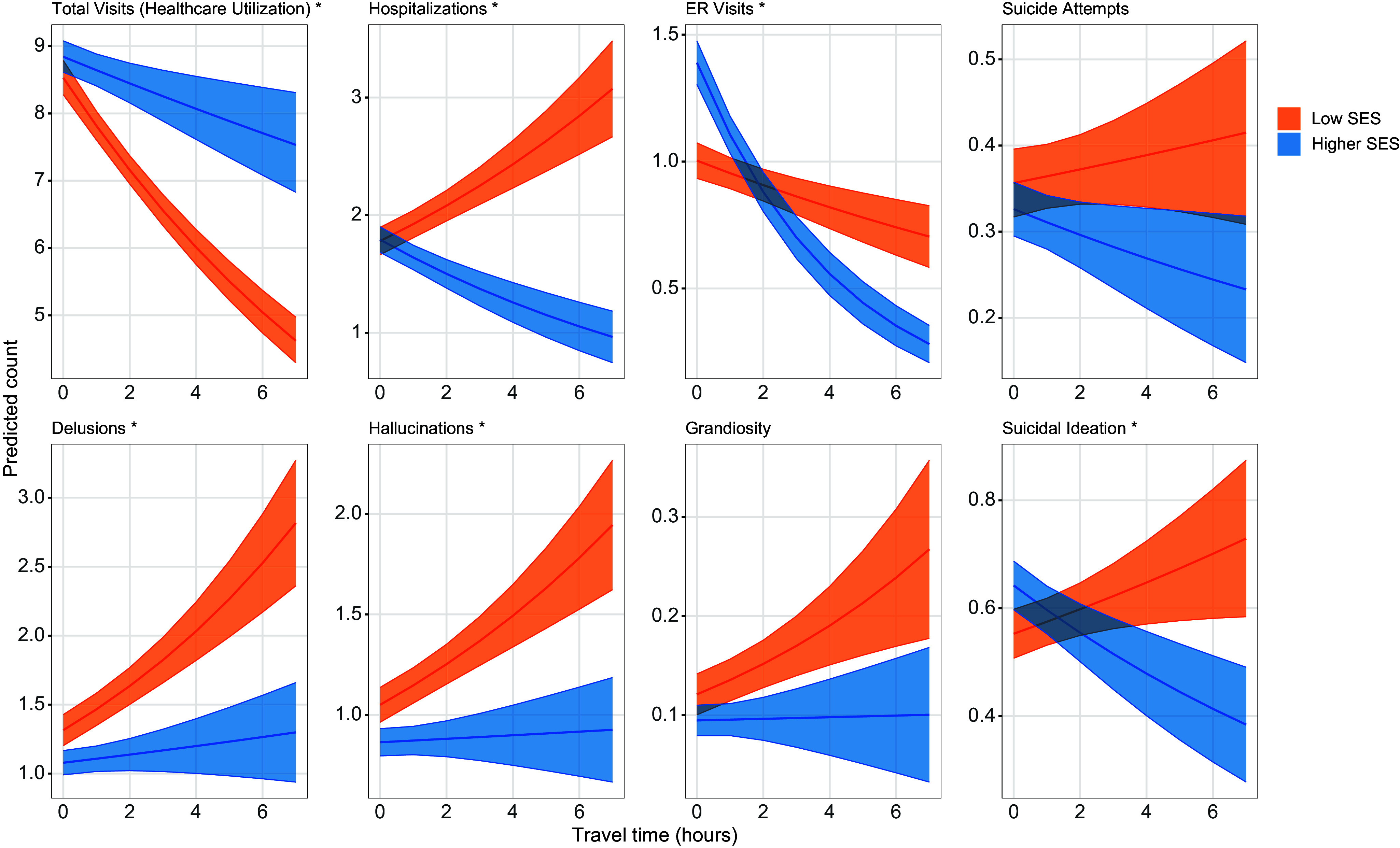
Interactions between travel time and socioeconomic status. Predicted counts of total visits (healthcare utilization), clinical outcomes, and symptoms are shown with 95% confidence intervals across increasing travel time. Predicted total visits were estimated using negative binomial regression, and outcome/symptom counts were estimated using zero-inflated negative binomial regression. Models with significant interactions, based on likelihood ratio tests with a Bonferroni correction threshold of 7.14e−03 (0.05/7 tests), are marked with an asterisk.

Patients with low SES had lower rates of healthcare utilization (total visits per year) with increasing travel time (LRT, *χ*^2^ = 47.98, df = 1, *P* = 4.30e−12), while utilization for higher SES patients remained stable (Figure 3).

Clinical outcomes and symptoms also varied by travel time and SES group. Low-SES patients living farther from the hospital had higher rates of hospitalization (LRT, *χ*^2^ = 72.53, df = 1, *P* = 1.65–17), a trend not observed among higher-SES patients. ER visits declined with increasing travel time for both groups, but more steeply among higher-SES patients (LRT, *χ*^2^ = 57.14, df = 1, *P* = 4.06–14). Low-SES patients with greater travel times also had higher rates of delusions and hallucinations, while symptom rates remained more stable across travel time among higher-SES patients. No significant interaction was found for suicide attempts or grandiosity. Full results from the omnibus testing are in Supplemental Table 3.

### Living with family moderates the effects of low SES

Within the cohort, 23% of patients lived with a partner and/or child/ren, 24% lived with parents, siblings, and/or extended family, 5% lived alone, and the remaining 48% patients had missing or uncategorizable household information (Supplemental Table 4). Patients diagnosed with BD and SCZ were more likely to live with parents, siblings, and/or extended family (BD: 26%; SCZ: 46%) compared to those diagnosed with MDD (19%). Missing household information was associated with low SES and living far from the hospital (Supplemental Table 5).

Briefly, patients living alone had 8% lower rates of utilization compared to those living with a partner and/or child/ren (RR, 0.92 [95% CI, 0.87–0.98], *P* = 6.92–03). Patients living alone also had higher rates of all clinical outcomes and symptoms. Patients living with parents, siblings, and/or extended family had similar rates of utilization compared to those living with a partner and/or child/ren. However, they had higher rates of hospitalization, suicidality, and grandiosity than those living with a partner and/or child/ren. See full details in Supplementary Tables 1 and 2.

**Figure 4.**
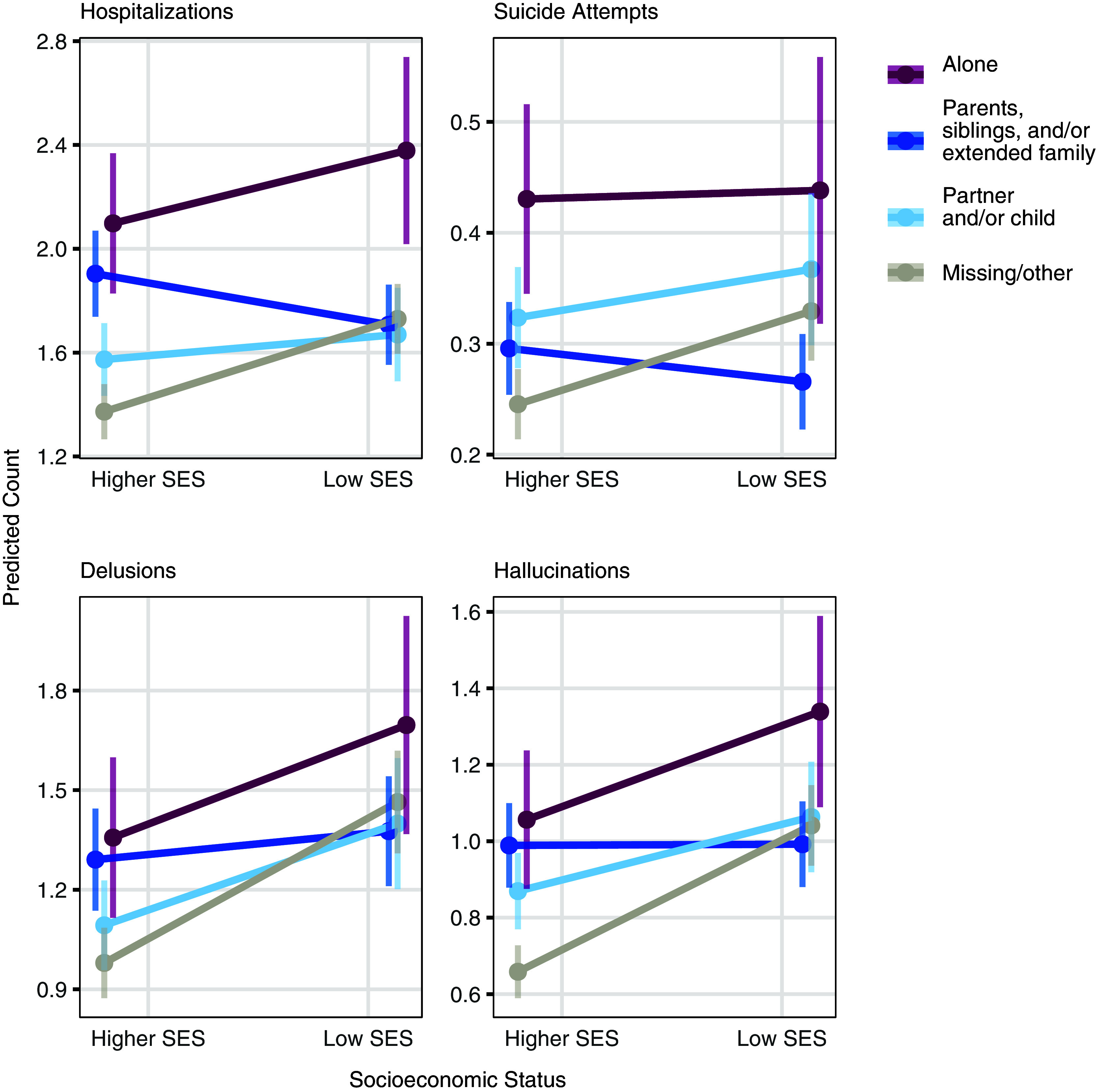
Interactions between household composition and socioeconomic status. Predicted counts of hospitalization, suicide attempts, delusions, and hallucinations, stratified by household composition and SES group. Predicted counts were estimated using zero-inflated negative binomial regression models and shown with 95% confidence intervals. Only significant results are shown based on likelihood ratio tests with a Bonferroni correction threshold of 7.14e−03 (0.05/7 tests).

Interestingly, the effect of SES on clinical outcomes/symptoms varied by household composition (Figure 4). Among patients living with family, rates of delusion and hallucination were similar across SES levels. However, low-SES patients living alone or with missing/other household data had significantly higher rates of both symptoms, compared to their higher-SES counterparts (delusions: LRT, *χ*^2^ = 27.75, df = 3, *P* = 4.10e−06; hallucinations: LRT, *χ*^2^ = 50.17, df = 3, *P* = 7.35e−11). A similar pattern was observed for hospital admissions and suicide attempts, where low-SES patients living with family had lower rates of both clinical outcomes (hospitalizations: LRT, *χ*^2^ = 47.08, df = 3, *P* = 3.35e−10; suicide attempts: LRT, *χ*^2^ = 19.51, df = 3, *P* = 2.14e−04). No significant SES interactions were found for other outcomes (Supplemental Figure 3, Supplemental Table 6). Travel time effects on clinical outcomes were largely driven by patients with missing/other information, with no significant differences among non-missing household groups (Supplemental Figure 4, Supplemental Table 7).

### Outpatient care usage associated with lower odds of adverse outcomes

From modeling visit-level changes, we found that having outpatient care in the last 2 months reduced the odds of all clinical outcomes and symptoms. Recent outpatient care had the largest effect on ER visits and suicidality, with an over 40% reduction in suicide attempts (OR, 0.54 [95% CI, 0.48–0.60], *P* = 6.23e−28) (Supplemental Table 8, Supplemental Figure 4). Conversely, having a hospitalization in the last 2 months increased the odds of all symptoms and clinical outcomes besides suicide attempts.

SES changes (both from low to higher and vice versa) were associated with increased odds of hospitalization, an ER visit, and suicidal ideation. Transitions to low SES were also associated with increased risk of suicide attempt, delusion, and hallucination. The visit-level effects of current SES and travel time on clinical outcomes and symptoms aligned with the effects from the patient-level models (Supplemental Table 2).

## Conclusions

Although previous studies have identified gaps in healthcare for individuals with severe psychiatric disorders in Colombia (Kohn et al., 2018), their impact on patient outcomes was understudied (McGinty & Eisenberg, 2022). Leveraging comprehensive EHRs from Clínica San Juan de Dios Manizales, our analyses revealed marked differences in both clinical outcomes and service utilization associated with socioeconomic status and travel time.

Consistent with prior research, BD and SCZ diagnoses were more prevalent among low-SES patients than in the general population (Agerbo et al., 2015; Campbell et al., 2022; Hakulinen et al., 2019; Kivimäki et al., 2020; Schoeyen et al., 2011), while low-SES patients were underrepresented among those with MDD. Given the established association between low SES and increased risk of MDD (Hoveling, Liefbroer, Schweren, Bültmann, & Smidt, 2022; Lasserre et al., 2022), our findings suggest that low SES may serve as a barrier preventing individuals with MDD from seeking treatment at CSJDM. These findings highlight the importance of examining barriers to care across diagnostic categories.

Unlike most prior studies in Latin America, which have relied on cross-sectional data and therefore could not assess changes over time (Barrios et al., 2022; Báscolo et al., 2020; Garcia-Ramirez et al., 2020; Garcia-Subirats et al., 2014; Kohn et al., 2018; Roberti et al., 2024), our longitudinal approach reveals that low-SES patients accessed care less frequently and often presented with more severe symptoms or required hospitalization when they did seek care.

Although some literature suggests that individuals with lower SES may experience more severe psychiatric symptoms, independent of treatment access, (Campbell et al., 2022; Islam & Adnan, 2017; Liu, Liu, Liang, & Luo, 2022), our findings indicate a more context-dependent relationship. Among patients living close to the hospital, clinical outcomes and symptom severity were similar across SES groups. Differences in rates of symptoms and adverse outcomes became more apparent with increasing travel time, suggesting that geographic barriers may delay care for low-SES individuals, resulting in more advanced illness by the time they receive treatment. These associations persisted after adjusting for educational attainment, suggesting that education may not be strongly confounding these relationships. However, unmeasured factors, such as baseline illness severity, genetic predisposition, comorbidities, or health/insurance literacy, may still contribute to the observed associations between travel time/SES and the outcomes.

Beyond differences in access and clinical outcomes, we also observed distinct patterns in how patients used specific types of services. For example, higher-SES patients living near the hospital were more likely to use emergency care compared to low-SES patients, contrasting with usage trends in North American settings (Giannouchos, Kum, Foster, & Ohsfeldt, 2019; Khan, Glazier, Moineddin, & Schull, 2011; S.-Y. Lee et al., 2022; Stern, Weissman, & Epstein, 1991). Further research is needed to understand the demographics of individuals frequently using emergency services under Colombia’s healthcare system.

Social/family support also appeared to influence care access and outcomes. Notably, the relationship between low SES on access to and outcomes of care was less pronounced for patients with family support. Patients living with family may have greater assistance in accessing care, navigating the healthcare system, or adhering to treatment, which could help reduce the severity of illness at the time of care.

Given these findings, it is important to emphasize the critical role of outpatient care in psychiatric treatment. Outpatient visits are vital for psychiatric care, enabling symptom monitoring and early intervention to reduce the risk of hospitalization and suicide (Fontanella et al., 2020; S. Y. Lee et al., 2015; Marcus, Chuang, Ng-Mak, & Olfson, 2017; Okumura, Sugiyama, & Noda, 2018). Our analysis showed that recent outpatient care was associated with lower odds of severe symptoms, hospital admissions, and suicide attempts; however, these models were neither causal nor clinical predictors. Rather, our findings may reflect a trend where patients who regularly attended outpatient visits were less likely to be hospitalized and/or were more likely to continue engaging in outpatient care.

Future research could focus on developing predictive models of suicide attempts and rehospitalization using clinical and demographic data. Additionally, studies should evaluate scalable interventions that enhance equitable access to care, such as telehealth and virtual consultations, to improve patient engagement, symptom management, and reduce readmissions. Since internet access is limited in rural Colombia, identifying high-risk areas is essential for guiding policymakers in allocating resources like mobile clinics and additional healthcare facilities.

Our work has several limitations. First, insurance was a practical but imperfect proxy for SES, as it cannot capture all the complexities of socioeconomic status. Similarly, household composition is only one facet of social support. Missing household data may also limit the generalizability of our findings, particularly for higher-SES and MDD patients. While the hospital’s EHR system is comprehensive, it does not account for patients who may seek care outside of CSJDM. A comprehensive understanding of psychiatric care access and health outcome disparities could be achieved by integrating the Registro Individual de Prestación de Servicios (RIPS), Colombia’s national registry of health service data (Bogotá Secretaría de Salud, *n.*d.; Ministerio de Salud y Protección Social, 2015) into EHR analyses.

In summary, this study identifies associations between SES, travel time, and mental healthcare access and outcomes, where low SES and longer travel times were linked to lower rates of healthcare utilization and higher rates of hospitalization and symptoms. Family support, however, may help patients better access care. These findings point to the potential value of targeted strategies to promote more equitable care for underserved populations.

## Supporting information

Gerdes et al. supplementary material 1 Gerdes et al. supplementary material

Gerdes et al. supplementary material 2 Gerdes et al. supplementary material

## Supplementary material

The supplementary material for this article can be found at http://doi.org/10.1017/S0033291725101694.
