# Supplementary material for: Socioeconomic and geographic disparities in psychiatric outcomes under Colombia’s universal healthcare system
Source: Psychol Med. 2025 Oct 13;55:e310. doi: 10.1017/S0033291725101694 (PMC12551587; doi:10.1017/S0033291725101694)
Supplement: Gerdes et al. supplementary material 1 — Gerdes et al. supplementary material [file S0033291725101694sup001.docx]

**Socioeconomic and geographic disparities in psychiatric outcomes under Colombia’s universal healthcare system**

Greta Gerdes^1^, Janet Song^1,2^, Susan Service^1^, Ana M Ramirez-Diaz^1^, Ana M Diaz-Zuluaga^1^, Alejandro Arias^3^, Mauricio Castaño-Ramirez^4^, Nicolas A. Crossley^3,5,6^, Carlos Lopez-Jaramillo^3^, Nelson Freimer^1^, Loes Olde Loohuis^1,7,8*^

1. Center for Neurobehavioral Genetics, Semel Institute for Neuroscience and Human Behavior, University of California Los Angeles, 760 Westwood Plaza, Los Angeles, CA 90024, USA
2. Northwestern University, Feinberg School of Medicine, 420 E. Superior St, Chicago, IL 60611, USA
3. Department of Psychiatry, University of Antioquía, Carrera 47 # 61-12, Ciudad Universitaria, Medellín, Colombia
4. Department of Mental Health and Human Behavior, Universidad de Caldas, Carrera 9a # 19-03, Manizales, Caldas, Colombia
5. Department of Psychiatry, University of Oxford, Warneford Hospital, Oxford, OX3 7JX, UK
6. Department of Psychiatry, Pontificia Universidad Católica de Chile, Diagonal Paraguay 362, Santiago, 8330077, Chile
7. Department of Human Genetics, David Geffen School of Medicine, University of California Los Angeles, 695 Charles E Young Dr S, Los Angeles, CA 90095, USA
8. Department of Computational Medicine, David Geffen School of Medicine, University of California Los Angeles, 10833 Le Conte Ave, Los Angeles, CA 90095, USA

**Supplemental Materials**

**Supplemental Methods**

**Supplemental Figure 1**. Comparison of socioeconomic distributions between patient and Caldas population.

**Supplemental Figure 2.** Sensitivity analysis: effects of SES on clinical outcomes and symptoms, stratified by residence in and outside of Manizales.

**Supplemental Figure 3.** Additional interactions between housing composition and socioeconomic status.

**Supplemental Figure 4.** Interactions between housing composition and travel time.

**Supplemental Figure 5.** Effects of recent care and changes in SES on visit-level outcomes and symptoms.

**Supplemental References**

**Supplemental Methods**

**Address formatting for geocoding pipeline**

Before geocoding the addresses, we standardize their format to align with the Colombian postal system. Most road systems in urban areas of Colombia are structured in a grid format with streets called “carreras” running North-South and streets called “calles” running East-West. If the residence is located on a carrera, the postal system formats the address as “Carrera [X] #[Y]-[Z], [Municipality], Caldas, Colombia”, where X is the street number of the carrera, Y is the street number of the calle, and Z refers to the specific building and the approximate number of meters from the intersection of streets X and Y. If the residence is located on a calle, the address is formatted as “Calle [Y] #[X]-[Z], [Municipality], Caldas, Colombia”, with X, Y, and Z representing the same numbers as mentioned previously. For addresses located in rural areas, we use address information exactly as it is listed in the EHR and include the municipality and department name. To account for potential errors in the geocoding procedure, we verify that the longitude and latitude coordinates provided by the geocoding API are within the boundaries of the municipality specified in the EHR for each address.

**Statistical model information**

All statistical models were performed in R. All models with **covariates (C)** listed include age, sex, diagnosis, and presence of a diagnostic switch. For models with bootstrap-generated, normal 95% confidence intervals (CIs) and two-sided p-values, 1,200 bootstrap iterations are used to create the distribution. Equation is listed under the model name.

1. **Comparing the SES distribution of patients to the general population**

- Exact binomial model
- Bonferroni correction threshold
  - When testing per municipality, the threshold is set to 2.00E-3 (0.05/25 tests), corresponding to the 25 municipalities in Caldas where patients reside.

1. **Modeling the effect of travel time and socioeconomic status on healthcare utilization, clinical outcomes, and symptoms**
   1. **Healthcare utilization**

number of visits ~ travel time + SES + log(hospitalization count +1) + offset(log(years in EHR))

- - - covariates = household composition + age + sex + diagnosis + diagnosis switch (binary) + year of diagnosis
    - Negative binomial regression
    - Modeling visits as a rate (visits/year) using the offset
    - Bonferroni correction threshold: 2.50E-2 (0.05/2 tests)
      - 1 outcome x 2 predictors of interest (travel time + SES)
    - CIs and p-values calculated from bootstrap distribution
  1. **Clinical outcomes and symptoms**

outcome/symptom count ~ travel time + SES + C + offset(log(number of visits)) | log(number of visits)

- - - covariates = household composition + age + sex + diagnosis + diagnosis switch (binary) + year of diagnosis
    - Zero-inflated negative binomial regression
    - Modeling outcomes/symptoms as a rate (instances/ # of visits) using the offset
    - Logit model using number of visits as a predictor
    - Bonferroni correction threshold: 3.57E-3 (0.05/14 tests)
      - 7 outcomes/symptoms x 2 predictors of interest (SES + travel time)
    - CIs and p-values calculated from bootstrap distribution
  1. **Sensitivity analyses**
     - **Modeling the effect of urban residence**
       - Models run separately for individuals in the Manizales municipality zone (urban) and for those in the remaining Caldas municipalities (rural)
       - outcome/symptom count ~ travel time + SES + C + offset(log(number of visits)) | log(number of visits)
       - covariates = household composition + age + sex + diagnosis + diagnosis switch (binary) + year of diagnosis
       - model run separately for individuals in urban Manizales municipality zone and for those in the remaining Caldas municipalities
       - CIs and p-values calculated from bootstrap distribution
     - **Modeling whether educational attainment impacts estimated effects of SES and travel time**
       - Same models as modeling approaches 2.1 and 2.2, but included educational attainment as an additional covariate

1. **Modeling travel time–SES interactions**
   1. **Healthcare utilization**

number of visits ~ travel time x SES + log(hospitalization count +1) + offset(log(years in EHR))

- - - covariates = household composition + age + sex + diagnosis + diagnosis switch (binary) + year of diagnosis
    - Likelihood ratio test for presence of significant interaction
    - No Bonferroni correct (only one test)
  1. **Clinical outcomes and symptoms**

outcome/symptoms count ~ travel time x SES + offset(log(number of visits)) | log(number of visits)

- - - covariates = household composition + age + sex + diagnosis + diagnosis switch (binary) + year of diagnosis
    - Likelihood ratio test for presence of significant interactions
    - Bonferroni correction threshold: 7.14E-3 (0.05/7 tests)
      - 7 outcomes/symptoms x 1 interaction

1. **Modeling interaction effects of household composition with SES and travel time**
   1. **Modeling patterns of missing household composition (HC) data in the EHR**

Presence of HC data (binary) ~ travel time + SES + age + sex + diagnosis + Dx year

- - - Logistic regression model
    - CIs and p-values calculated from bootstrap distribution
  1. **Household composition – SES interactions**
     - **Healthcare utilization**

Number of visits ~ HC x SES + log(hospitalization count +1) + offset(log(years in EHR))

- - - - covariates = travel time + age + sex + diagnosis + diagnosis switch (binary) + year of diagnosis
      - Likelihood ratio test for presence of significant interaction
    - **Clinical outcomes and symptoms**

outcome/symptoms counts ~ HC x SES + offset(log(number of visits)) | log(number of visits)

- Likelihood ratio test for presence of significant interaction
- Bonferroni correction threshold: 7.14E-3 (0.05/7 tests)
  1. **Household composition – travel time interactions**
     - **Healthcare utilization**

Number of visits ~ HC x travel time + log(hospitalization count +1) + offset(log(years in EHR))

- - - - covariates = SES + age + sex + diagnosis + diagnosis switch (binary) + year of diagnosis
      - Likelihood ratio test for presence of significant interaction
    - **Clinical outcomes and symptoms**

outcome/symptoms counts ~ HC x travel time + offset(log(number of visits)) | log(number of visits)

- - - - covariates = SES + age + sex + diagnosis + diagnosis switch (binary) + year of diagnosis
      - Likelihood ratio test for presence of significant interaction
      - Bonferroni correction threshold: 7.14E-3 (0.05/7 tests)

1. **Modeling visit-to-visit variability in clinical outcomes and symptoms**

Outcome/symptoms (binary) ~ SES change (since last visit) + Hosp Last 2 Mo + Outpatient Last 2 Mo + (1 | EHR_ID + year)

- - - covariates = SES (at visit) + age (at visit) + sex + diagnosis (at visit) + diagnostic change (since last visit)
    - Mixed effect logistic regression
    - Bonferroni correction threshold: 1.79E-3 (0.05/28 tests)
      - 7 outcomes/symptoms x 4 longitudinal predictors of interest (two types of SES changes, prior hospitalizations, and prior outpatient visits)

**R packages for statistical analysis**

1. MASS v7.3.60 (Venables & Ripley, 2003)
   - Negative binomial regression models
2. pscl v1.5.9 (Achim Zeileis, Kleiber, & Jackman, 2008)
   - Zero-inflated negative binomial regression model
3. glmmTMB v1.1.10 (Brooks et al., 2017)
   - mixed-effects logistic regression models
4. emmeans v1.8.9 (Searle, Speed, & Milliken, 1980)
   - estimated marginal means for interaction models
5. lmtest v0.9.40 (A. Zeileis & Hothorn, 2002)
   - likelihood ratio test
6. boot v1.3-31 (Davison & Hinkley, 1997)
   - boostrap p-values and 95% confidence intervals

**Supplemental Figures**

**Supplemental Figure 1. Comparison of socioeconomic distributions between patient and Caldas population.** Results from exact binomial tests are shown per municipality per diagnosis, with color scale indicating difference in percent of individuals with low SES between the patient and Caldas population. Bonferroni correction threshold = 2.00E-3 (0.05/25 municipalities).


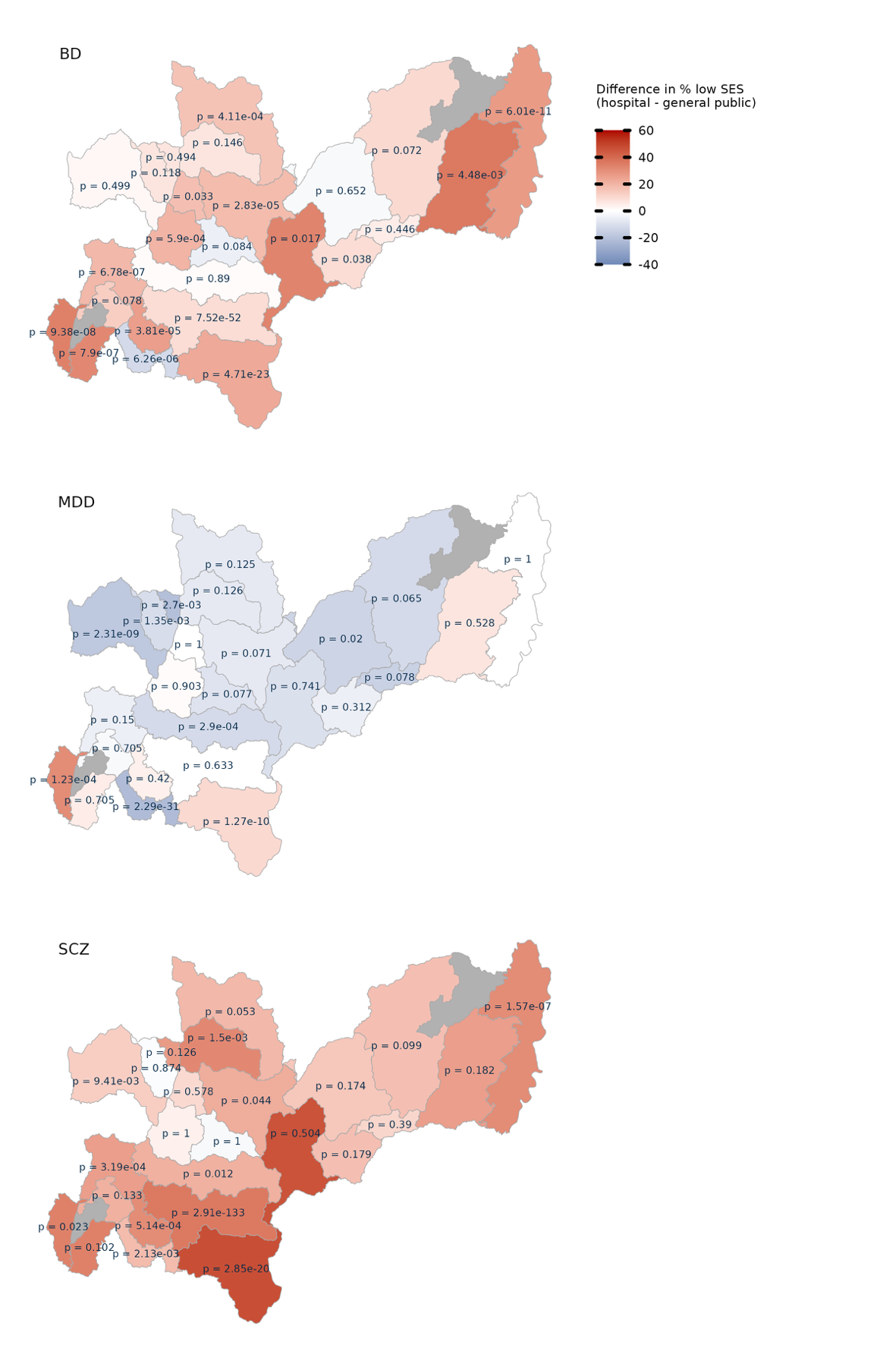


**Supplemental Figure 2. Sensitivity analysis: effects of SES on clinical outcomes and symptoms, stratified by residence in and outside of Manizales.** Rate ratios from zero-inflated negative binomial regression models are shown with bootstrap 95% confidence intervals.

**
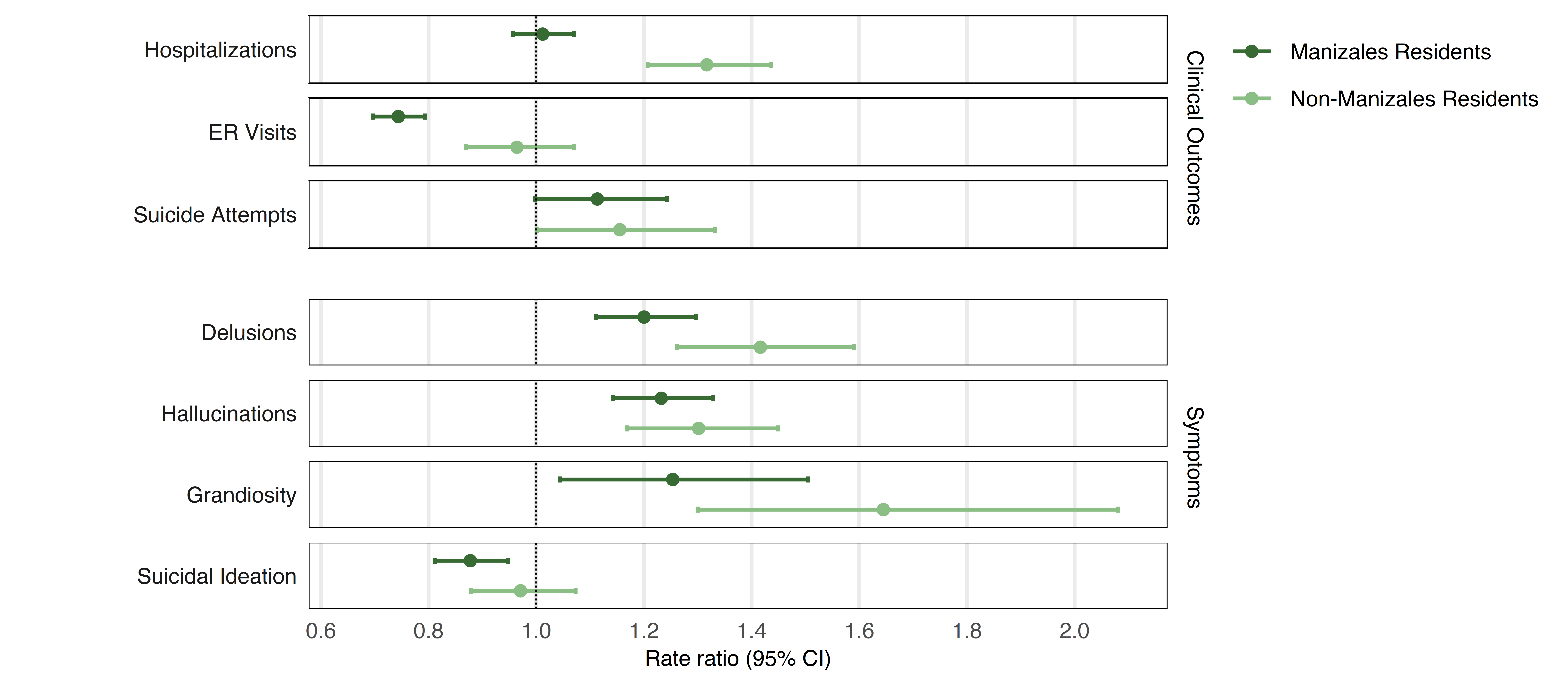
**

**Supplemental Figure 3. Additional interactions between housing composition and socioeconomic status.** Predicted counts of total visits (healthcare utilization), ER visits, and mentions of grandiosity and suicidal ideation, are shown with 95% confidence intervals and stratified by household composition and SES group. Predicted counts were estimated using zero-inflated negative binomial regression models and shown with 95% confidence intervals. Models with significant interactions, based on likelihood ratio tests with Bonferroni correction threshold of 7.14e−03 (0.05/7 tests), are marked with an asterisk.

**
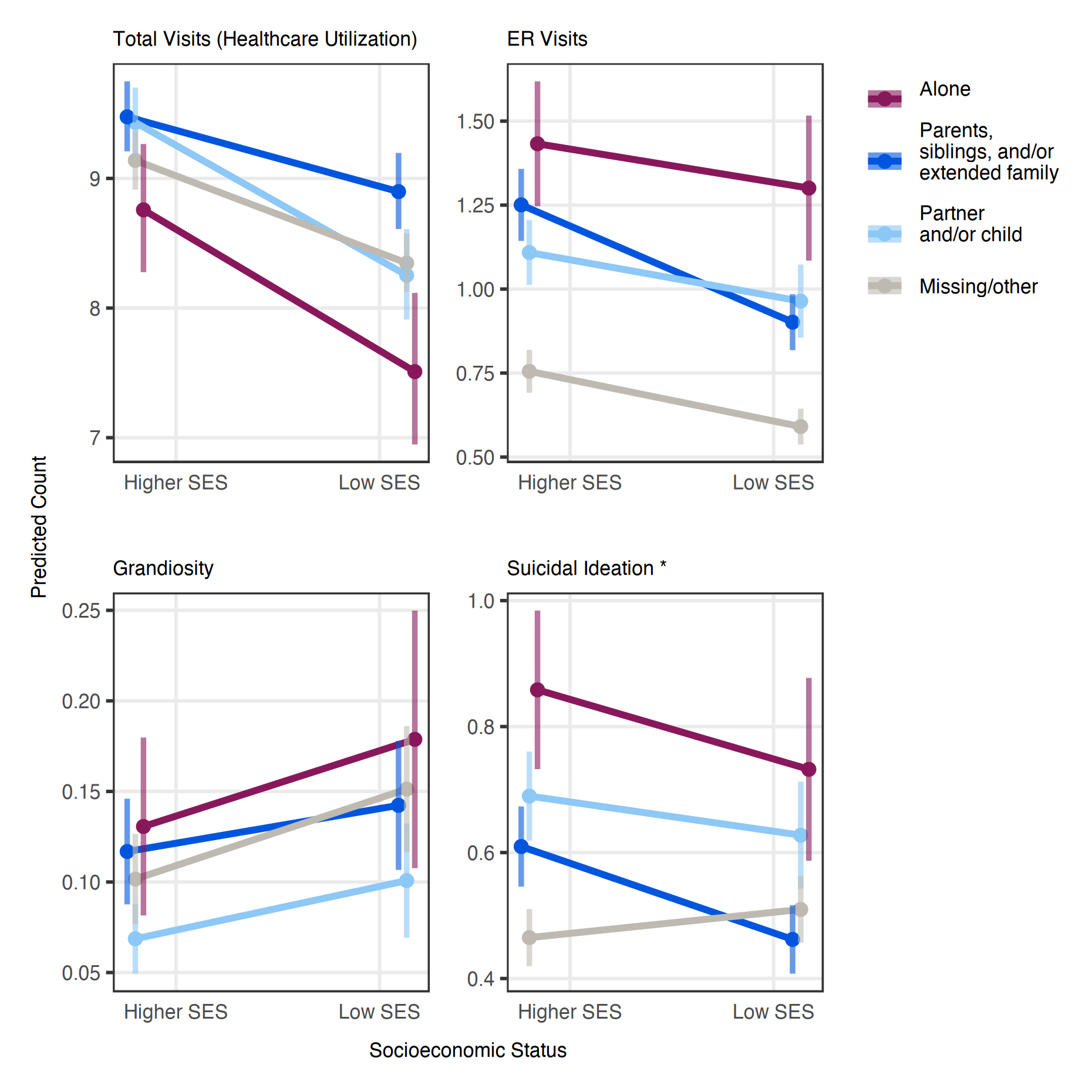
**

**Supplemental Figure 4. Interactions between housing composition and travel time.** Predicted counts of total visits (healthcare utilization), clinical outcomes, and symptoms are shown with 95% confidence intervals for household composition groups across travel time. Clinical outcomes/symptoms are estimated using zero-inflated negative binomial regression models, while total visits are estimated using a negative binomial regression model. Models with significant interactions, based on likelihood ratio tests with Bonferroni correction threshold of 7.14e−03 (0.05/7 tests), are marked with an asterisk.

**
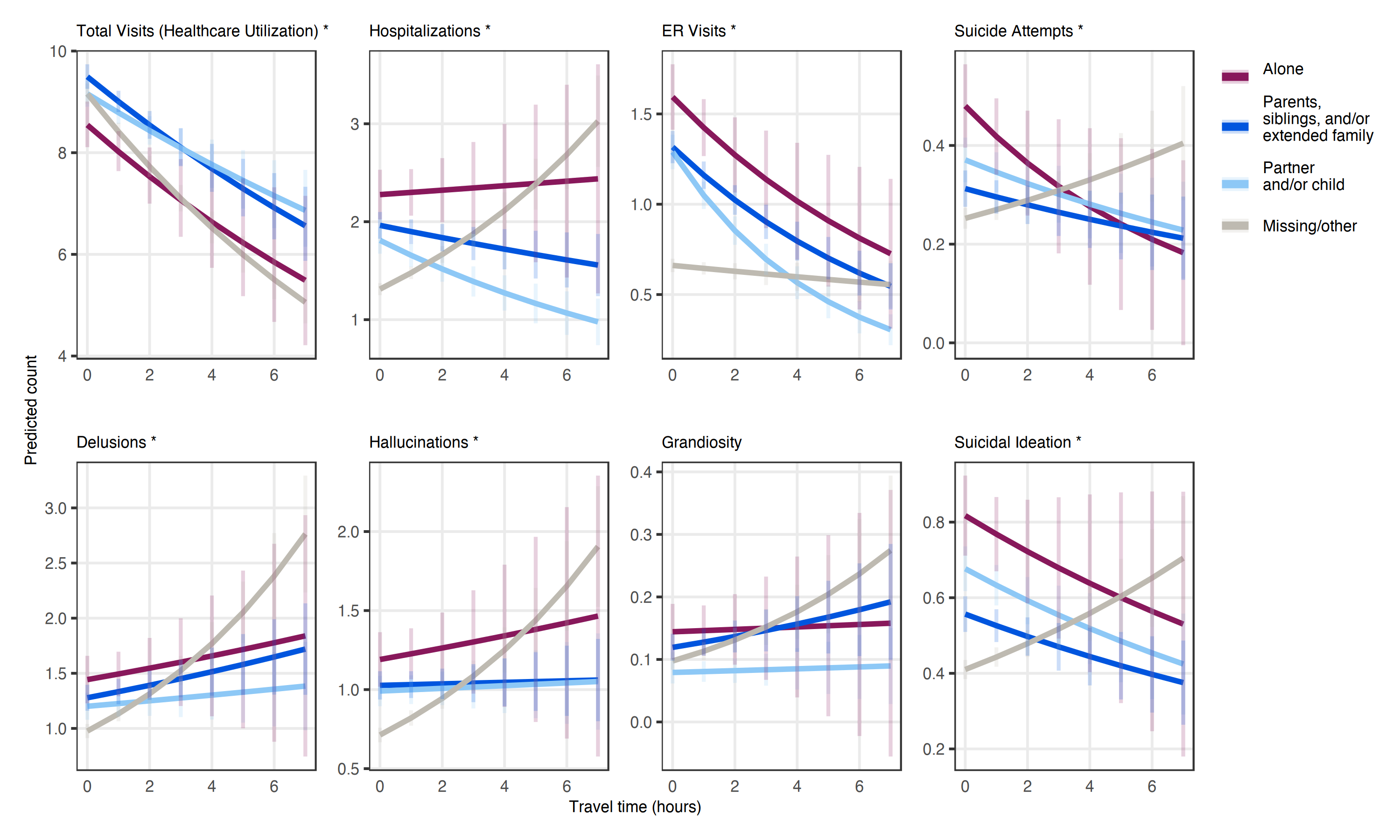
**

**Supplemental Figure 5. Effects of recent care and changes in SES on visit-level outcomes and symptoms.** Odds ratios from mixed-effects logistic regression models are shown with 95% confidence intervals. Significant results, based a Bonferroni correction threshold of 1.79E-3 (0.05/28 tests), are highlighted in red.

**
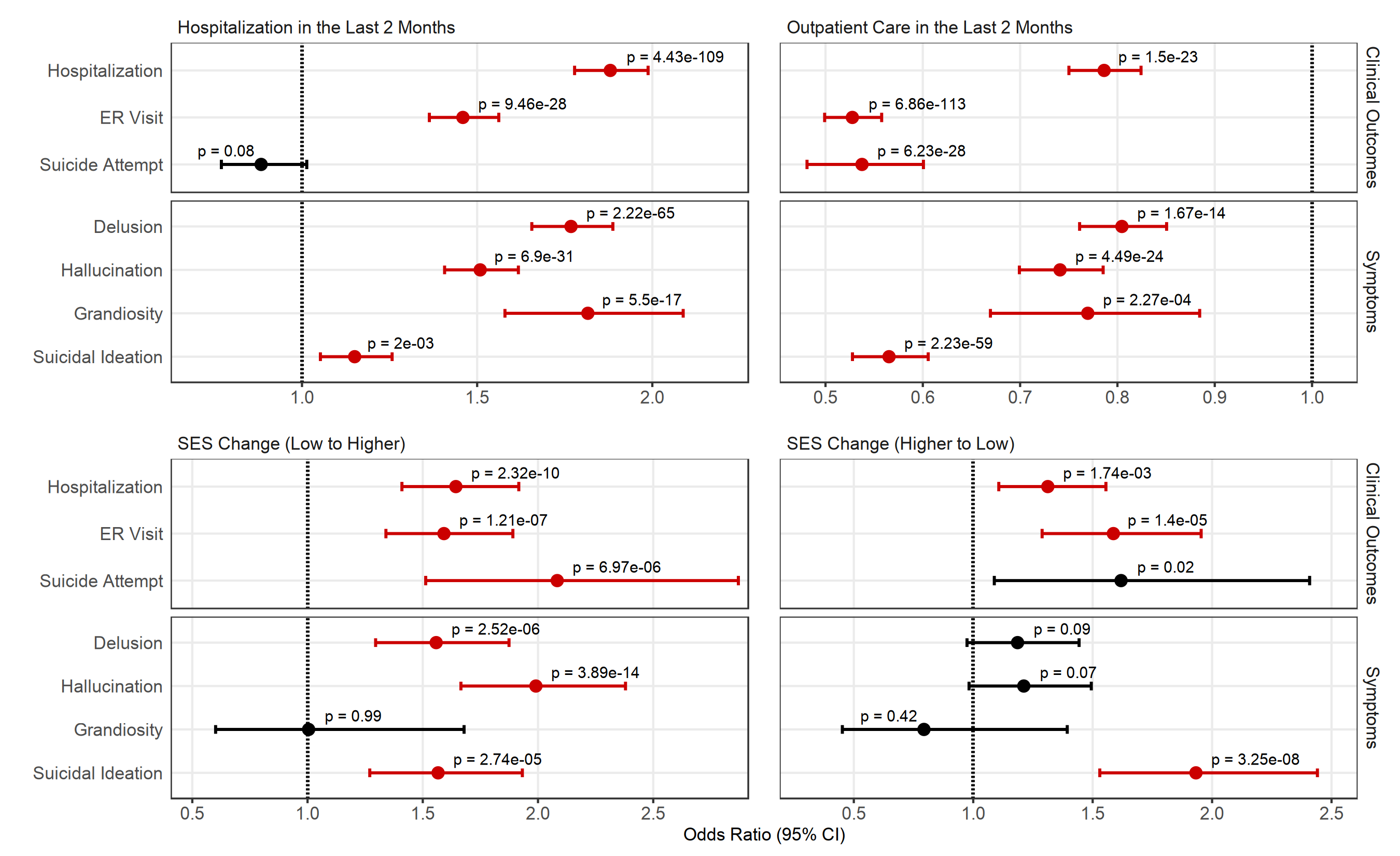
**

**Supplemental References**

Brooks, M., Kristensen, K., Benthem, K. van, Magnusson, A., Berg, C., Nielsen, A., … Bolker, B. (2017). GlmmTMB balances speed and flexibility among packages for zero-inflated generalized linear mixed modeling. *The R Journal*, 9(2), 378. Retrieved from https://doi.org/10.32614/rj-2017-066

Davison, A. C., & Hinkley, D. V. (1997). *Bootstrap methods and their application*. Cambridge University Press. Retrieved from https://doi.org/10.1017/cbo9780511802843

Searle, S. R., Speed, F. M., & Milliken, G. A. (1980). Population marginal means in the linear model: An alternative to least squares means. *The American Statistician*, 34(4), 216–221. Retrieved from https://doi.org/10.1080/00031305.1980.10483031

Venables, W. N., & Ripley, B. D. (2003). *Modern Applied Statistics with S*. Springer Science & Business Media. Retrieved from https://play.google.com/store/books/details?id=974c4vKurNkC

Zeileis, A., & Hothorn, T. (2002). Diagnostic Checking in Regression Relationships. *R News*, 2(3), 7–10.

Zeileis, A., Kleiber, C., & Jackman, S. (2008). Regression models for count data inR. *Journal of Statistical Software*, 27(8), 1–25. Retrieved from https://doi.org/10.18637/jss.v027.i08
